# Different Arbuscular Mycorrhizal Fungi Established by Two Inoculation Methods Improve Growth and Drought Resistance of Cinnamomum Migao Seedlings Differently

**DOI:** 10.3390/biology11020220

**Published:** 2022-01-29

**Authors:** Xuefeng Xiao, Jingzhong Chen, Xiaofeng Liao, Qiuxiao Yan, Gelin Liang, Jiming Liu, Deng Wang, Ruiting Guan

**Affiliations:** 1 Forestry College, Research Center of Forest Ecology, Guizhou University, Guiyang 550025, China; jovenxiao@163.com (X.X.); chengjingzhong-2016@foxmail.com (J.C.); yanqxecho@sina.com (Q.Y.); green523000@163.com (G.L.); wangdengyaya@163.com (D.W.); GRTyoum21@163.com (R.G.); 2 Institute of Mountain Resources, Guizhou Academy of Science, Guiyang 550001, China; lxfnsd@163.com

**Keywords:** AMF, afforestation, antioxidant enzyme, *Cinnamomum migao*, climate change, drought stress, Karst region, osmotic adjustment

## Abstract

**Simple Summary:**

Drought is a global climatic phenomenon and one of the main factors that negatively affect plant growth. Karst is a unique type of ecosystem where ecological degradation is becoming more and more serious due to the aggravation of global drought. Vegetation restoration is an effective method for preventing ecological degradation in Karst ecosystems. *Cinnamomum migao* is selected as the tree species for vegetation restoration, because it is a unique, fast-growing medicinal plant of Southwest China that only thrives in Karst regions. Arbuscular mycorrhizal fungi (AMF) are an important component of the soil biota in ecosystems and alleviate drought stress in plants by forming a mutualistic symbiosis. Most previous studies just considered the effects of AMF species on drought resistance but did not evaluate different inoculation methods. The aim of the present study was to compare the effects of different AMF resulting from the use of different inoculation methods on the growth and drought resistance of *C. migao* seedlings in Karst soil. The findings of this study will improve the success rate of reforestation programs in Karst ecosystems through the utilization of these important microorganisms.

**Abstract:**

Drought stress is one of the greatest obstacles affecting field crop productivity in arid and semi-arid regions, and its severity and frequency are expected to increase due to human-induced changes to the environment and climate. Drought has led to rocky desertification in Karst regions. *Cinnamomum migao* is a unique, fast-growing medicinal plant of Southwest China that only thrives in Karst regions. Arbuscular mycorrhizal fungi (AMF) symbiosis alleviates drought stress in plants; however, establishment and function of the symbiotic interaction between AMF host plant in relation to the inoculation method remain unclear. Therefore, we conducted an experiment to investigate the effects of AMF species (*Glomus etunicatum* and *Funneliformis mosseae*) and two inoculation methods (seed vs. seedling inoculation) under drought stress on *C. migao* seedlings, and quantified mycorrhizal colonization, AMF spore density, root vigor, relative water content, *C. migao* growth, antioxidant enzyme activities, and osmotic adjustment. Inoculation with AMF (*G. etunicatum* and *F. mosseae*) positively affected the growth and root vigor of *Cinnamomum migao* under drought stress, regardless of the inoculation method. Additionally, both AMF species markedly upregulated antioxidant enzyme activities and osmotic adjustment substances, regardless of the inoculation method. Our results showed that the collective stimulatory effect of *G. etunicatum* is more efficient than that of *F. mosseae*. AMF application could promote afforestation with *C. migao* to prevent rocky desertification in Karst regions where water is the greatest limiting factor on plant growth and yield.

## 1. Introduction

Drought stress is a global climatic phenomenon and one of the main factors that has negative socioeconomic and ecological effects, as well as greatly hinders plant growth [1] and crop production through changes in physiology [2,3]. The severity and frequency of drought are expected to increase in the coming century [4,5]. Drought stress greatly limits plant productivity in Karst ecosystems, which are deteriorating. Karst is a unique type of ecosystem characterized by thin soils and poor nutrients that is distinguished by its diverse landforms, which originated from limestone, dolomite, and carbonate rocks [6], and spans 22.00 million km^2^, covering 15% of the Earth’s land surface [7]. Shallow soils, exposed bedrock, and its underground drainage system have led to high surface temperatures and aggravated drought stress [8]. The largest area of Karst rocky desertification is in Guizhou Province of Southwest China [9]. Due to the aggravation of global drought stress, ecological degradation in Karst landforms is becoming increasingly more serious and leading to a sharp decrease in surface vegetation coverage and soil erosion, known as rocky desertification [10]. Recent studies have shown that rocky desertification is detrimental to soil quality and plant diversity [11,12].

Vegetation restoration is an effective method for preventing ecological degradation in Karst ecosystems, and appropriate species for afforestation are the most critical and important factors [13,14]. *Cinnamomum migao* is a tree species used for vegetation restoration, which was determined through our preliminary literature review, field visits, and field ecological environment surveys. *C. migao* is a unique medicinal plant found in Southwest China that only exists in the dry-hot valleys of the transitional zone in Karst regions of the Guizhou, Guangxi, and Yunnan Provinces of China. It is an important industrial raw material and spice, as well as a traditional medicine with a long history in the Miao nationality [15]. Medicine made from this plant generates hundreds of millions of yuan every year. *C. migao* is a fast-growing tree species that is distributed across the Karst ecosystem and has great economic value [15]. Unfortunately, in recent years, its wild resource reserve has rapidly declined due to extensive and unregulated logging, and its natural renewal disorder.

To improve the drought resistance and survival rate of *C. migao* seedlings in Karst regions, arbuscular mycorrhizal fungi (AMF), which are beneficial microorganisms in the soil [16], are considered in this study as they are associated with the roots of most plants, stimulate plant growth, and enhance plant tolerance to abiotic stress [17]. These traits allow AMF to form symbiotic relationships with plant roots, in which the fungus receives carbon from its host plant and in turn provides nutrients to the plant [18], thereby improving host plant tolerance to biotic and abiotic stressors, such as drought [19,20,21]. AMF influence several mechanisms of plant stress tolerance, such as morphological adaptations, osmotic adjustment, optimization of water resources, and reactive oxygen species (ROS) [22,23]. More recently, the growth of AMF in inoculated plants before afforestation under drought conditions has been observed in the Karst regions of China [24]. *Glomus* is recognized as a dominant AMF genus in the Karst regions of Southwest China [25]. Furthermore, previous research has confirmed that *C. migao* can co-exist with some AM fungi [26,27], and recently a study has analyzed the effects of AMF on *C. migao* drought tolerance under water deficit conditions in Karst soils by assessing inoculated seedlings [28]. However, none has explored the effects of different AMF inoculation methods or synergistic interaction between AMF species and inoculation methods on *C. migao* performance.

A previous study indicated that different inoculation methods could have significantly different effects on tomato yield [29]. However, it is not known whether the inoculation method affects the AMF symbiosis.

The aim of this research is to fill a gap in the existing literature on the use of AMF in *C. migao* plant growth, antioxidant enzyme activities, and osmotic regulation compounds under drought stress, and to explore a more appropriate and useful protocol for afforestation with *C. migao* inoculated by AMF in Karst regions. We hypothesized that: (i) AMF inoculation can positively affect the growth and drought resistance of *C. migao* under drought stress conditions, and (ii) the synergistic interaction between AMF species and inoculation method will positively affect *C. migao* plant growth and drought resistance.

We compared the effects of two AMF species (*G. etunicatum*) and (*F. mosseae*) applied by two methods of inoculation (seed vs. seedling inoculation), and measured growth performance, antioxidant enzyme activities, and osmotic regulation compounds under drought stress. The findings of this study should be considered to improve the success rate of reforestation programs in Karst ecosystems.

## 2. Materials and Methods

### 2.1. Plant Seeds, AMF, and Soil

*C. migao* seeds were collected from healthy adult trees in the Karst forest of Guizhou Province, China, in October 2019. Similar-sized seeds were selected, surface sterilized with 5% NaClO for 10 min, and washed 5 times with sterile distilled water. Seeds were germinated in 200 g sterilized sand (121 °C for 4 h) in plastic seed trays in a temperature incubator (25 °C, 80% relative humidity). Seedlings were irrigated with sterile water once a day before transplanting into pots.

*G. etunicatum* and *F. mosseae* were used as the fungal inocula, which were obtained from the Beijing Academy of Agriculture and Forestry Sciences, Beijing, China. The initial spore concentration of the inocula was 120/g. Before experimentation, both inocula were propagated in sterile potting soil using host white clover (*Trifolium repens*). After 4 months, the spore density was determined by the wet sieve method [30]. The fungal inoculum was obtained from the white clover rhizosphere, including a mixture of spores (70/g), infected root fragments, and soil.

Soil was collected from Karst areas with severe rocky desertification that will undergo afforestation (25°64′ N, 107°07′ E; elevation: 790.28 m), including three barren hills and four plots of abandoned lands. Each barren hill was divided into 3 parts according to the altitude, and then 60 bags of soil samples were randomly collected from each part with “S-route” in the horizontal direction (approximately 1 kg of soil per bag). Similarly, four abandoned lands were divided symmetrically into 16 blocks of 20 m × 20 m, and then 40 bags of soil samples were randomly collected from each block with “S-route”. The surface litter and 2 cm of topsoil were removed when collecting soil at a depth of about 20 cm. There were 1180 bags (approximately 1180 kg) soil in total which were mixed together evenly before sterilization. The soil had a pH of 7.13 ± 0.31 (measured in water, 1:5 *w/v*), 24.06 ± 1.40 g kg^−1^ organic matter, 13.61 ± 0.82 g·kg^−1^ organic carbon, 3.30 ± 0.13 g kg^−1^ total N, 0.99 ± 0.05 g kg^−1^ total P, 16.02 ± 1.80 g kg^−1^ total K. The soil samples were sieved (2 mm) and mixed with sand (1–2 mm) and perlite (2–4 mm), (5:1:1, soil: sand: perlite, w: w: w) (121 °C, 0.14 MPa for 4 h). Then, 4 kg sterilized mixed soil was used to fill the pots (22.5 cm diameter × 22.5 cm height).

### 2.2. Study Site

This study was performed in a plastic greenhouse (average temperature: 18.7 °C·y^−1^, mean humidity: 55%, average illumination: 1148.3 h·y^−1^) located at Guizhou University in Guiyang City, Southwest China (26°340′ N, 106° 420′ E; elevation: 242–1020 m). The site has a subtropical monsoon climate with an annual mean temperature of 20.35 °C. The average annual rainfall is 1200 mm with 235 d of rainfall and 1148 h of sunshine.

### 2.3. Experimental Design

The experiment tested the effects of two factors: (1) mycorrhizal infection (*G. etunicatum*, *F. mosseae*, and non-AMF (NM)) and (2) inoculation methods (inoculation of seed (M-seed) and inoculation of seedlings (M-seedlings)). There were six treatments in total. Each treatment had 60 replicates (pots) resulting in a total of 360 pots. Pots were randomly placed in the outdoor greenhouse. No fertilizer was used during the experiment.

In a pilot study, the germination rates of the *C. migao* seeds were uneven, therefore, an excess amount of 1000 seeds had been prepared.

For the seed inoculation method (M-seed), 500 sterilized seeds were planted in the center of sterilized sand (121 °C for 4 h) in plastic seed trays, at the same time, 20 g (dry wt.) mycorrhizal inoculum (soil, spores, hyphae, and root material) was placed 5 cm under the sand surface so that it was in direct contact with seeds. The control treatment contained 20 g autoclaved mixed inoculum. A total of 10 kg inoculum was used. After 3 months of germination, 180 similar-sized seedlings with sterilized sand in plastic seed trays were transplanted to pots, one plant per pot, giving a total 180 pots.

The seedling inoculation method (M-seedling method) consisted of 500 sterilized seeds that were planted in the center of sterilized sand in plastic seed trays for germination. After 3 month of germination, 180 similar-sized seedlings with sterilized sand in plastic seed trays were transplanted to pots, at the same time, 20 g of inoculum was placed 5 cm under the soil surface so that it was in direct contact with the seedling roots. The control treatment contained 20 g autoclaved mixed inoculum. Similarly, one plant per pot was transplanted giving a total 180 pots.

The study was conducted from November 2019 to January 2021. Seeds were put in plastic seed trays in November 2019. In February 2020, seedlings were transplanted to pots. After 60 d of growth, mycorrhizal colonization and AMF spore density per 10 g soil were dynamically measured every 60 d until the end of the experiment. Plants were well-watered during the first 300 d every 2 d. The drought treatment was applied at d 300 with natural evaporation until the end of the experiment. During drought stress, soil moisture, the relative water content (RWC) of plant leaves, and root vigor were dynamically measured every 5 d. After 315 d of growth when the top of the leaves of most plants were wilting, all plants were harvested and other indicators were measured.

### 2.4. Parameters Measured

For sample collection and processing, six pots were randomly selected from each treatment.

#### 2.4.1. Mycorrhizal Colonization

Six plants were randomly selected from each treatment and at each sampling time. A total of 200 root segments which were cut to 1 cm from the middle part of the root for each treatment, and cleaned with 10% (*w/v*) KOH for 30 min at 90 °C, then stained with 0.05% (*w/v*) trypan blue in lactophenol [31]. Stained root tissues were examined under a light microscope (CX43, Olympus, Tokyo, Japan). AM colonization was quantified according to the following formula [32]:

AMF colonization (%) = (root length colonized/total root length observed) × 100

#### 2.4.2. AMF Spore Density Per 10 g Soil

Six plants were randomly selected from each treatment at each sampling time. Then, 3 cm of surface soil was removed from the pots. After removing large soil particles from the root system, the firmly attached soil (1–2 mm from the root tissue) was collected with a total of 10 g, which was regarded as the rhizosphere soil [33] Then, samples were wet sieved and the decantation-sucrose centrifugation method was used to separate soil AMF fungal spores [34]. The number of spores was recorded under a light microscope (CX43, Olympus, Tokyo, Japan).

#### 2.4.3. Root Vigor

Six plants were randomly selected from each treatment at each sampling time. Fresh root tip was cut into about 2 cm segments in each treatment, cleaned with sterile distilled water, and weighed to approximately 1 g, and then soaked in 0.4% TTC and phosphate buffer for 1–3 h at 37 °C in the dark. Then, 1 mol/L H_2_SO_4_ was added for 1 min. Afterwards, root segments were wiped dry and grinded [35,36].

Root vigor(mg·g^−1^·h^−1^) = TTC reduction (mg)/Root weight(g)·T(h)

#### 2.4.4. RWC

The RWC of the fifth full and fresh leaves were measured following previously described methods [2]:

RWC (%) = (FW − DW)/(SW − DW) × 100

where FW is the fresh weight, DW is the dry weight, and SW is the saturated weight of the leaves.

#### 2.4.5. Soil Moisture

Soil moisture was measured using a portable soil temperature, moisture, salinity rapid measuring instrument (TY-WSY, TengYuYi, Zhengzhou, China) with 15 cm long probes that estimated the depth of the root zone.

#### 2.4.6. Plant Growth and Mycorrhizal Dependency Index

A steel ruler was used to measure plant height, a vernier caliper was used to measure the base and taproot diameters, and a leaf area scanner (LI-COR Biosciences, Lincoln, NE, USA) was used to measure the leaf area index. Harvested seedlings were separated into roots and aboveground parts, then dried at 85 °C for 30 min, and oven-dried at 70 °C to a constant dry weight. The mycorrhizal dependency index (MDI) was calculated using the following formula [37]:

MDI (%) = (DW of AMF plants − DW of NM plants)/DW of AMF plants × 100. (NM plants are the plants without AMF inoculation)

#### 2.4.7. Antioxidant Enzyme Activity

Six plants were randomly selected from each treatment at each sampling time, and then the fifth functional leaf from top was collected from each plant.

Frozen leaves (0.5 g) were extracted with ice-cold 50 mM phosphate buffer (pH = 7.8). The homogenates were centrifuged and the supernatant was used to analyze superoxide dismutase (SOD) and catalase (CAT) activities. SOD activity was measured by nitroblue tetrazolium (NBT) at 560 nm according to [38]. CAT activity was assayed by measuring the decrease in absorbance at 240 nm due to H_2_O_2_ decomposition.

A total of 2.0 g frozen leaves were extracted with ice-cold 50 mM phosphate buffer (pH = 5.5). The homogenates were centrifuged and the supernatant was used to analyze peroxidase (POD) enzymes. POD activity was measured using the guaiacol method [35]. Then, 1.0 g frozen leaves were grinded with 10% (*w/v*) trichloroacetic acid (TCA). The homogenates were centrifuged and the supernatant was boiled with 0.6% (*w/v*) thiobarbituric acid (TBA) for 15 min, which was for measuring the malondialdehyde (MDA) content. Then, the boiled supernatant was measured at 600 nm (A600), 532 nm (A532), and 450 nm (A450). The MDA content was calculated using the following equation:

C = 6.45 × (A532 − A600) − 0.56 × A450

#### 2.4.8. Osmotic Adjustment Substances

Six plants were randomly selected from each treatment at each sampling time, and then the fifth functional leaf from top was collected from each plant.

Fresh leaves (1 g) were grinded in 5% (*w/v*) sulfosalicylic acid to obtain free proline (Pro), which was measured by spectrophotometric analysis at 515 nm using the ninhydrin reaction [39]. Then, 1 g fresh leaves were grinded in 80% (*v/v*) alcohol to obtain the total soluble sugars (SS), which were analyzed by 0.1 mL anthrone. The absorbance at 620 nm was determined in a spectrophotometer (Hitachi U-1900; Hitachi Corporation, Tokyo, Japan). The concentration of soluble protein (SP) was determined following the method using Coomassie brilliant blue G-250 with bovine serum albumin as the standard at 595 nm [40].

### 2.5. Statistical Analysis

All data are presented as the average of 6 replications of each treatment. Data were statistically analyzed using a two-way analysis of variance (ANOVA) with SPSS v21.0 (IBM, Armonk, NY, USA). Then, significant (*p* < 0.01, *p* < 0.05) treatment differences were verified via Tukey’s post hoc test. Percentage values were arcsine transformed prior to the analyses. All graphs were produced using OriginPro v9.0 (Origin Lab, Northampton, MA, USA).

## 3. Results

### 3.1. AMF Colonization and Spore Density

**Figure 1:**
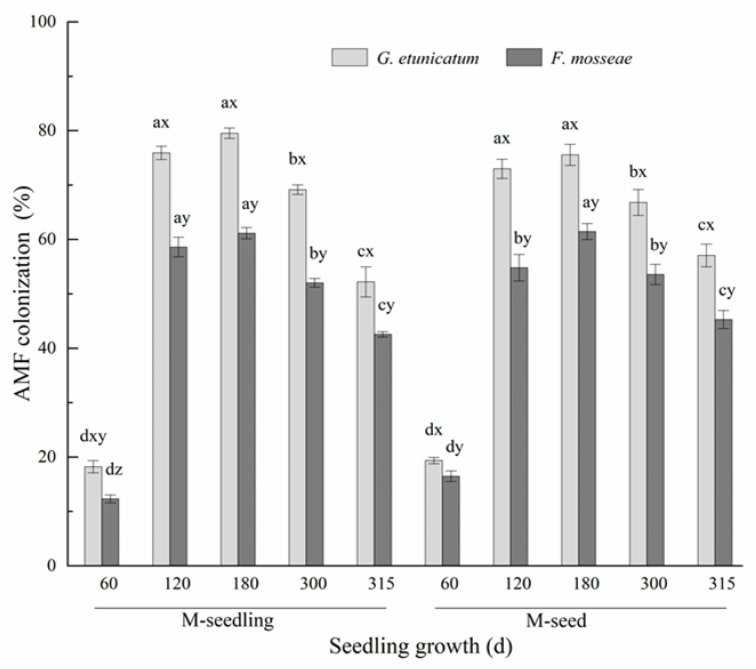
Root colonization rate of *C. migao* seedlings under 6 different treatments (the root colonization rate of NM was 0). Different lowercase letters (x, y, z) in the same d of seedling growth (*x*-axis) indicate the result of Tukey’s post hoc test at the 95% confidence level between treatments. Other different letters (a, b, c, d) indicate significant differences between different stages under the same treatment (*p* < 0.05). NM, non-AMF plants; *G. etunicatum*, plants inoculated with *G. etunicatum*; *F. mosseae*, plants inoculated with *F. mosseae*; M-seedling, inoculated by the seedling method; M-seed, inoculated by the seed method. Values are expressed as the mean ± SE (*n* = 6, which are treatment replicates).

We observed vesicles, arbuscules, and hyphae in the inoculated roots (Figure S1). AMF colonization under different treatments increased at first and then decreased (Figure 1), and reached the lowest point (12.31–18.22% in M-seedling; 16.47–19.37% in M-seed) at d 60 and the highest point (61.15–79.51% in M-seedling; 61.45–75.56% in M-seed) at d 180 after inoculation. These findings show that AMF had not completely formed a symbiotic relationship with the roots by d 60. AMF colonization was almost unchanged from d 120 to 180, and then decreased after the drought treatment began. AMF colonization by *G. etunicatum* was always markedly higher than *F. mosseae*, regardless of the inoculation method. AMF colonization was significantly affected by the inoculation method only in the initial stage of inoculation (60 d), showing higher values in plants emerging from M-seed compared to M-seedling method. However, as plants grew, this difference gradually disappeared.

**Figure 2:**
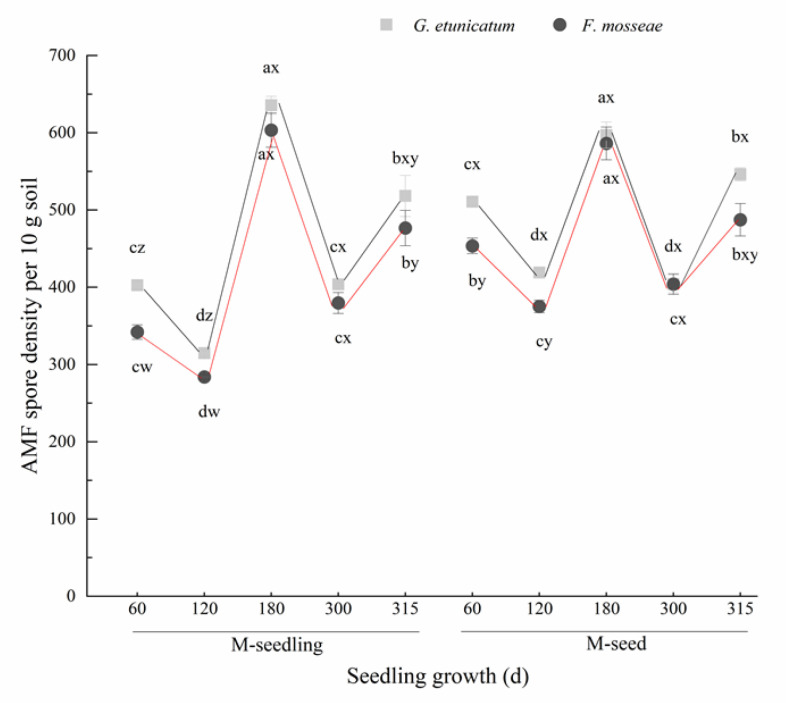
AMF spore density per 10 g soil of *C. migao* seedlings under 6 different treatments (there are no AMF spores of NM). Different lowercase letters (w, x, y, z) in the same d of seedling growth (*x*-axis) indicate the results of Tukey’s post hoc test (*p* < 0.05) between treatments. Other different letters (a, b, c, d) indicate significant differences between different stages under the same treatment (*p* < 0.05). NM, non-AMF plants; *G. etunicatum*, plants inoculated with *G. etunicatum*; *F. mosseae*, plants inoculated with *F. mosseae*; M-seedling, inoculated by the seedling method; M-seed, inoculated by the seed method. Values are expressed as the mean ± SE (*n* = 6, which are treatment replicates).

The spore density in the rhizosphere soil showed a “W” change trend as plants grew, regardless of the AMF status or inoculation method (Figure 2), and reached the lowest point at d 120, increased at mid-inoculation, and peaked at d 180. The spore density in plants inoculated with M-seed method was always higher than with M-seedling method at the onset of inoculation (60–120 d), but no difference was detected by the time of late-decomposition (180–315 d).

### 3.2. Root Vigor and RWC

**Figure 3:**
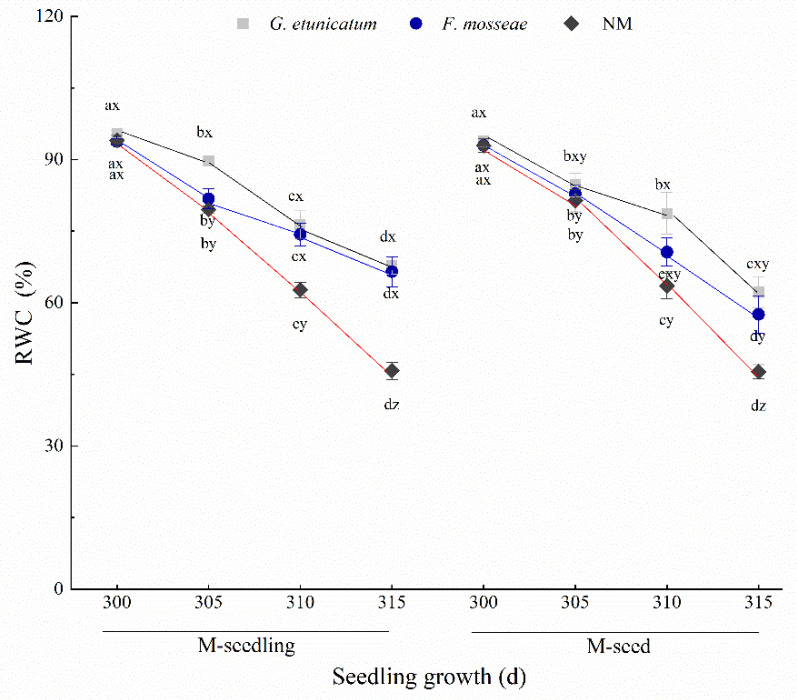
Root vigor of *C. migao* seedlings under 6 different treatments. Different lowercase letters (x, y, z) in the same d of seedling growth (*x*-axis) indicate the results of Tukey’s post hoc test (*p* < 0.05) between treatments. Other different letters (a, b, c, d) indicate significant differences between different stages under the same treatment (*p* < 0.05). NM, non-AMF plants; *G. etunicatum*, plants inoculated with *G. etunicatum*; *F. mosseae*, plants inoculated with *F. mosseae*; M-seedling, inoculated by the seedling method; M-seed, inoculated by the seed method. Values are expressed as the mean ± SE (*n* = 6, which are treatment replicates).

We sequentially measured root vigor every 5 d after the drought treatment began. The root vigor of each treatment was the highest at d 300 and decreased afterwards (Figure 3), but this result was not significant. The root vigor of nonmycorrhizal (NM) plants markedly decreased and was the lowest at every growth stage. Likewise, root vigor also decreased in AMF plants. However, the root vigor of AM plants especially in *G. etunicatum* plants was still higher than that of NM plants during the experiment. Moreover, there was no detectable effect on root vigor due to inoculation method.

**Figure 4:**
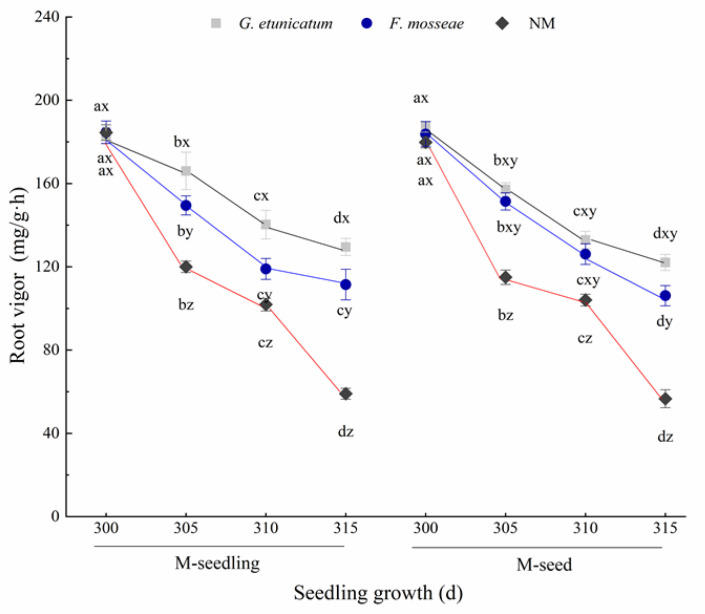
Relative water content (RWC) of *C. migao* seedlings under 6 different treatments. Different lowercase letters (x, y, z) in the same d of seedling growth (*x*-axis) indicate the results of Tukey’s post hoc test (*p* < 0.05) between treatments. Other different letters (a, b, c, d) indicate significant differences between different stages under the same treatment (*p* < 0.05). NM, non-AMF plants; *G. etunicatum*, plants inoculated with *G. etunicatum*; *F. mosseae*, plants inoculated with *F. mosseae*; M-seedling, inoculated by the seedling method; M-seed, inoculated by the seed method. Values are expressed as the mean ± SE (*n* = 6, which are treatment replicates).

The RWC of leaves is the earliest and most direct manifestation of plant reactions to drought stress and reflects the water deficit state of plants in adverse conditions [41]. The RWC among different treatments before drought stress was similar, at approximately 90% (Figure 4). However, drought stress unevenly reduced the RWC among treatments, and was seriously reduced in NM plants, where it reached the lowest point after drought treatment began. By contrast, the RWC of AMF plants only slightly declined in both inoculation methods (M-seedling 27.70% and 29.17%; M-seed 33.7% and 38.08%).

### 3.3. Plant Growth and MDI

**Figure 5:**
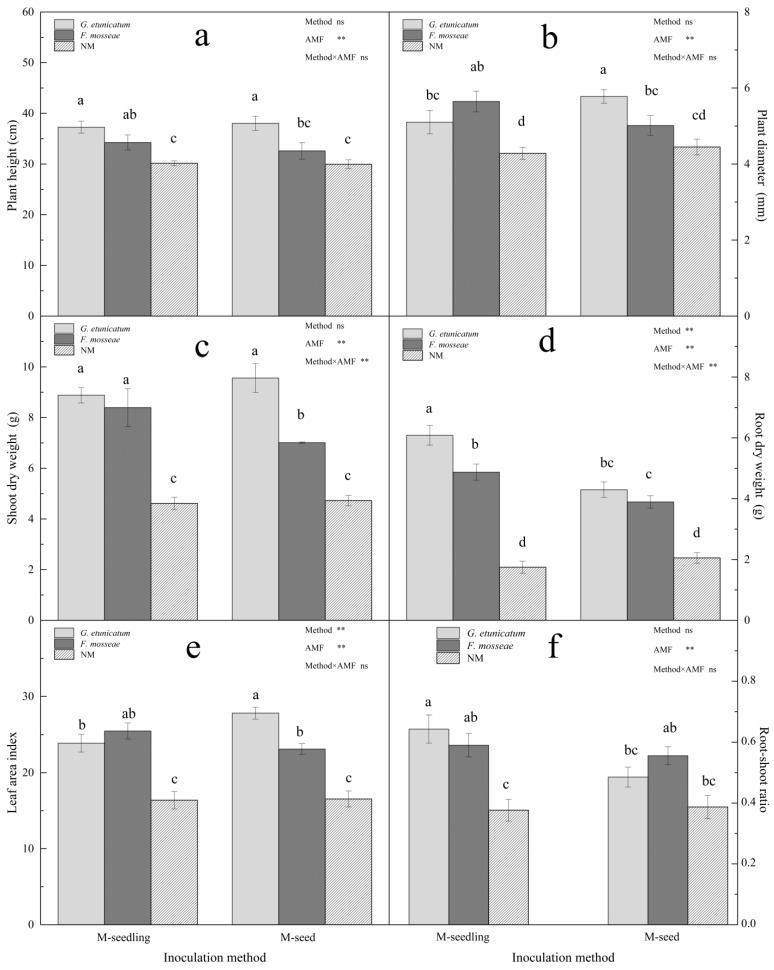
Plant growth of *C. migao* seedlings under 6 different treatments. (**a**) Plant height of *C. migao* seedlings under 6 different treatments, (**b**) Plant diameter of *C. migao* seedlings under 6 different treatments, (**c**) Shoot dry weight of *C. migao* seedlings under 6 different treatments, (**d**) Root dry weight of *C. migao* seedlings under 6 different treatments, (**e**) Leaf area index of *C. migao* seedlings under 6 different treatments, (**f**) Root-shoot ratio of *C. migao* seedlings under 6 different treatments. Different letters (a, b, c, d) indicate a significant difference of Tukey’s post hoc test ( ** *p* < 0.01; ns, not significant) between all treatments. NM, non-AMF plants; *G. etunicatum*, plants inoculated with *G. etunicatum*; *F. mosseae*, plants inoculated with *F. mosseae*; M-seedling, inoculated by the seedling method; M-seed, inoculated by the seed method. Values are expressed as the mean ± SE (*n* = 6, which are treatment replicates).

After 15 d of drought stress, the experiment was terminated, plants were harvested, and plant growth indicators were measured. Plant height and stem diameter were significantly lager in all inoculated seedlings under drought stress (Figure 5a,b), regardless of inoculation method. The plant height emerging from the M-seedling method increased by 23.58% and 13.59% after inoculation with *G. etunicatum* and *F. mosseae*, respectively (Figure 5a). Similarly, the plant height emerging from the M-seed method increased by 26.89% and 8.74% after inoculation with *G. etunicatum* and *F. mosseae*, respectively. The stem diameter emerging from the M-seedling method significantly increased by 32% after inoculation with *F. mosseae* (Figure 5b). The stem diameter emerging from the M-seed method inoculated with *F. mosseae* increased significantly, by 29.89%. Clearly, AMF status significantly (*p* < 0.01) affected plant height and stem diameter, indicating that *G. etunicatum* was more effective than *F. mosseae*, while inoculation method had no effect.

The shoot and root dry weights of AMF seedlings were markedly greater than NM seedlings under drought stress (Figure 5c,d). When compared to the NM treatment, *G. etunicatum* inoculation significantly increased shoot dry weight by 92.42% when emerging from the M-seedling method, and *F. mosseae* inoculation increased by 81.6%. When emerging from the M-seed method, the dry weight of *G. etunicatum* inoculation increased by 100% when compared to the NM treatment (Figure 5c). AMF inoculation increased root dry weight under both inoculation methods (Figure 5d). When emerging from the M-seedling method, the root dry weight significantly increased by 249.43% and 179.89% after inoculation with *G. etunicatum* and *F. mosseae*, respectively. Likewise, when emerging from the M-seed method, the root dry weight markedly increased by 109.29% and 89.76% after inoculation with *G. etunicatum* and *F. mosseae*, respectively. These results showed that the inoculation method and AMF status both significantly affected the leaf area index (Figure 5e). When compared to the NM treatment, the leaf area index of AMF plants significantly increased by 68.18% and was the greatest in *G. etunicatum* plants when emerging from the M-seed method. When emerging from the M-seedling method, the AMF status had no significant effect on the leaf area index. Additionally, the root-shoot ratio markedly increased after AMF inoculation and was the greatest in *G. etunicatum* plants, regardless of inoculation method (Figure 5f). AMF status significantly affected the root-shoot ratio, while the inoculation method did not.

**Figure 6:**
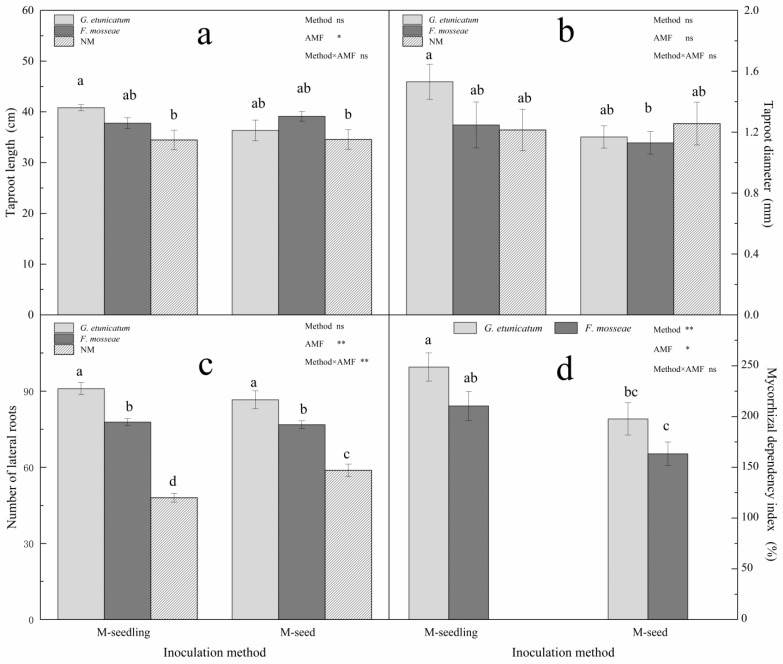
Root system and mycorrhizal dependency index (MDI) of *C. migao* seedlings under 6 different treatments. (**a**) Taproot length of *C. migao* seedlings under 6 different treatments, (**b**) Taproot diameter of *C. migao* seedlings under 6 different treatments, (**c**) Number of lateral roots of *C. migao* seedlings under 6 different treatments, (**d**) mycorrhizal dependency index of *C. migao* seedlings under 4 different treatments. Different letters (a, b, c, d) indicate a significant difference of Tukey’s post hoc test (* *p* < 0.05; ** *p* < 0.01; ns, not significant) between all treatments. NM, non-AMF plants; *G. etunicatum*, plants inoculated with *G. etunicatum*; *F. mosseae*, plants inoculated with *F. mosseae*; M-seedling, inoculated by the seedling method; M-seed, inoculated by the seed method. Values are expressed as the mean ± SE *(n* = 6, which are treatment replicates). * *p* < 0.05; ** *p* < 0.01; ns, not significant.

AMF colonization positively affected the taproot length and number of lateral roots, but not the taproot diameter (Figure 6 and Figure S5). Two-way ANOVA revealed significant independent effects of AMF status on taproot length, but no effects due to method or their interactions (Figure 6a). *G. etunicatum* colonization had the greatest positive effect on taproot length. Two-way ANOVA revealed that the number of lateral roots was significantly affected by AMF status and the interaction between AMF status and inoculation method (Figure 6c). There was a strong significant positive effect due to AMF status (*p* < 0.01). Moreover, when compared to NM, *G. etunicatum* colonization increased the number of lateral roots by 89.58% when inoculated by the M-seedling method. MDI is an estimation of plant responses to mycorrhizal colonization in terms of biomass enhancement [42]. The results revealed that MDI was significantly affected by AMF status and inoculation method individually, but not by their interactions (Figure 6d). Growth differences were more evident in AMF plants inoculated by the M-seedling method, where the MDI values were 248% and 210% higher under drought stress after colonization with *G. etunicatum* and *F. mosseae*, respectively.

### 3.4. Antioxidant Enzyme Activity

Although there was no significant difference in the MDA content between inoculation methods, inoculation significantly decreased the MDA content when compared to the control under drought stress (Table 1). The mean MDA content decreased by 55.26% and 47.85% after inoculation with *G. etunicatum* and *F. mosseae*, respectively, when inoculated by the M-seedling method, and decreased by 49.72% and 44.43% when inoculated by the M-seed method, respectively. Two-way ANOVA revealed that only the AMF status had a significant effect on the MDA content, while inoculation method did not.

The results showed that the SOD, POD, and CAT activities in AMF plants were significantly greater when compared to NM plants under drought stress, regardless of inoculation method (Table 1). The SOD activity of AMF plants treated with *G. etunicatum* was the greatest among all treatments. Similarly, the POD activity of *G. etunicatum* plants was the greatest and increased (Table 1). The CAT activity of AMF plants treated with *G. etunicatum* was also the greatest among all treatments and increased by 288.53% and 245.06% when inoculated by the M-seedling and M-seed methods when compared to NM plants, respectively. Two-way ANOVA revealed significant independent effects of AMF status and inoculation method, and their interactions on SOD and POD activities, but not on CAT activity.

### 3.5. Osmotic Adjustment Substances

The Pro content of AMF plants was markedly lower than in NM plants, but the differences were not significant between *G. etunicatum* and *F. mosseae* under drought stress (Table 2). These results indicated that AMF inoculation reduced the accumulation of Pro in plants under drought stress. The Pro content of leaves was the lowest (0.61) in *F. mosseae* plants inoculated by the M-seedling method and decreased by 81.9% when compared to NM. No significant differences of the SP content between AMF and NM seedlings were detected. The SS content of leaves was greatly affected by AMF inoculation and was clearly greater in AMF plants than in NM plants. The SS content was the greatest in *G. etunicatum* plants (3.42) when inoculated by the M-seedling method and increased by 37.35% when compared to NM. Moreover, the SS content was the greatest in *F. mosseae* plants (3.13) when inoculated by the M-seed method and increased by 37.28% when compared to NM.

## 4. Discussion

Drought stress places an alarming abiotic constraint on current and future global agricultural production and rocky desertification [43], as well as adversely affects plant physiology, growth, and productivity [44]. Previous research has focused on the mechanisms by which plants flexibly adapt to these unfavorable conditions. One plant strategy is to establish AMF symbiosis and this symbiotic relationship is a key factor that helps plants cope with drought conditions [45,46].

### 4.1. AMF Colonization and Spore Density

Our study showed that the rate of mycorrhizal colonization was significantly reduced by drought stress, indicating that drought stress negatively affected the AMF colonization of plant roots. This phenomenon may be due to the inhibition of spore germination and hyphae extension by drought stress [47,48], which has been confirmed in *Poncirus trifoliata* [49], *Robinia pseudoacacia* [50], *Ceratonia siliqua* [51], and *Pelargonium graveolens* [52]. Moreover, stomata are sensitive to drought stress and close to maintain the internal water balance of plants, which weakens photosynthesis and thereby reduces the carbohydrate supply to AMF from the roots [53]. Species of genus *Glomus* are widely distributed in Karst areas [25,54]. In this study, *G. etunicatum* was isolated from a Karst region located in the Guizhou Province of Southwest China, where soils of our experiment were obtained. This AMF species exhibited higher colonization when compared to our second AM fungus (*F. mosseae*), regardless of whether or not plants were under drought conditions, suggesting that *G. etunicatum* is adapted to its native plant host.

Previous reports indicated that AMF spore density is mainly and simultaneously affected by its own germination, the phenological phase of host plants, and soil properties, and these factors often change with the seasons and climate [55]. In our study, AMF spore density exhibited a “W” trend and was the lowest at d 120 and highest at d 180, regardless of inoculation method or AMF status (Figure 2). The average temperature of the environment was 20 °C at d 120 (Figure S2), which is the optimal temperature for AMF spore germination [56]. As a result, fewer spores were detected in the rhizosphere soil. However, at d 180, the ambient temperature was as high as 26 °C and the spores went dormant to avoid damage. Additionally, the host plant provides carbohydrates to AMF and the demand for carbohydrates differs with growth stage [57]. There are two possible reasons why spore density significantly increased in the late stage of drought stress. One is that the temperature sharply dropped below 10 °C after 300 d, which made spores go dormant (Figure 2). The other is that the soil moisture was only 5% (Figure S4), which reduced photosynthesis and the nutrient absorption capacity of plants, thereby reducing the quantity of available carbohydrates that plants provide to AMF [53].

### 4.2. Root Vigor and RWC

Drought stress limits root growth, which results in reduced water and nutrient uptake [58]. Root vigor is a direct indicator of the effects of nutrient absorption and biomass accumulation. AMF inoculation can benefit the rhizosphere environment of roots and expand the root area through extra-root hyphae, as well as free enzymes, such as gloomycin, to gather soil water and maintain the permeability, water holding capacity of the rhizosphere soil [59], and higher root vigor.

Leaf RWC is the earliest and most direct manifestation of the response of above-ground plant parts to drought stress, reflecting the water deficit state of plants under abiotic stress [41]. Drought stress affects leaf RWC on both the cellular and whole plant levels, which leads to various specific and non-specific phenotypic and physiological responses [3]. In the present study, we found that the RWC of all treatments decreased during drought stress. This may be because RWC is related to cell turgor, enzyme activities, the decomposition, synthesis, and transportation of organic matter, and formation of new organs in plants [59]. The RWC of AMF plants was always higher than NM plants during the experiment, demonstrating that AMF may have a positive effect on enzyme activities and protect plant cells from turgor and damage caused by drought [32]. It is also possible that AMF can help plants absorb water.

Li et al. [60] cloned two aquaporin genes from *Rhizophagus intraradices* and performed functional verification, indicating that AMF can transport water to plants at the molecular level. Ruth et al. [61] quantified the contribution of water absorption by AMF to *Hordeum vulgaris* using a high-resolution moisture sensor, finding that the water absorbed by AMF hyphae accounts for 20% of the water absorbed by plants. Li et al. [62] found that AMF can offset and replace the role of fine roots. Under drought stress, the water absorption rate of AMF hyphae is 2–7 times greater than that of normal water [48].

### 4.3. Plant Growth

In our experiment, both AMF species enhanced plant growth, regardless of inoculation method (Figure S3). Specifically, *G. etunicatum* was more effective at improving plant growth under drought conditions when compared to *F. mosseae*. This difference may be due to *G. etunicatum* having a higher colonization rate, which enhanced *C. migao* growth and provided additional direct transport channels for water and mineral nutrients under drought conditions [63].

Root systems, especially the number of lateral roots and underground biomass (Figure S5), were the most significantly affected by AMF. This could be because AMF improved root plasticity, where plants expanded their absorption area by increasing the number of lateral roots, thereby obtaining more water and nutrients under drought stress [64]. The root-shoot ratio of AMF plants was markedly greater than in NM plants (Figure 6f), indicating that AMF plant biomass was transferred to the roots, which demonstrated that rich root systems are a mode by which plants resist drought stress [65]. Moreover, root length and lateral root density are positively increased by AMF [64,66], thereby improving the effective space allocation of the root system and optimizing its structure so that the root system has better access to water [59,67].

The root hydraulic conductivity of AMF plants is strengthened by the extensive AMF hyphal network in the soil, which provides an important pathway for extracting more water and nutrients [68], thereby increasing the biomass [64,69]. Previous reports indicated that AMF contribute significantly to the absorption of N, P, K, Fe, Mn, and Cu by plants to resist drought stress [70]. AMF plants release phytase and nitrate reductase [71,72], which affect the content of plant transporters that regulate the absorption of these minerals [73].

### 4.4. Antioxidant Enzyme Activity

Drought stress causes the overproduction of ROS, which results in cell membrane damage and eventually cell death in the plants [2], and plants that are protected against excess amounts of ROS [23,53] possess several antioxidant mechanisms, including enzymatic and nonenzymatic antioxidants. There are two reasons that explain why AMF relieve the oxidative damage of plants under abiotic stress. One is that AMF induce higher antioxidant enzyme activities as a protective mechanism to scavenge the generation of ROS in plants under drought stress [54]. Similarly, in our study we found that the SOD, POD, and CAT activities in AMF plants were greater than in NM plants. This result matches with similar findings in pistachio [74], *Cyclobalanopsis glauca* [54], and sunflowers [69]. Previous studies confirmed that the gene expressions of PtFe-SOD, PtMn-SOD, PtPOD, and PtCAT increased in AMF plants when compared to NM plants under drought conditions [49,50]. The second reason is that AMF hyphae help mycorrhizal plants absorb water immediately and abate drought stress, thereby avoiding the production of ROS [75,76]. MDA is a product of oxidative damage in plants and its content is positively correlated with the plasmonization and oxidation of plant cell membranes. In our study, the MDA content of AMF plants was notably lower than in NM plants, demonstrating that AMF protected plant cells from ionization. These results confirmed the role of arbuscular mycorrhizas in protecting host plants under unfavorable environmental conditions [77].

### 4.5. Osmotic Adjustment Substances

The accumulation of osmotic adjustment substances is a prominent characteristic of plants under drought resistance and maintains the balance of osmotic pressure and improves the water retention capacity [78]. Plant drought resistance is related to the accumulation of SS and Pro under drought conditions. In this study, the concentration of SS in AMF plants increased significantly when compared to NM plants. This result was consistent with the findings of [79], who reported that AMF plants actively accumulated some SS, thereby reducing the osmotic potential and freezing point. A previous study found that AMF significantly increased the sucrose, glucose, and fructose contents in the leaves of *Fructus aurantii* [80]. AMF have been extensively reported to increase the SP content of plants, which indicates that AMF infection may alleviate or decrease RNA disassembly and enhance the ability of the nonenzymatic antioxidant defense system by means of SP [81]. In our study, no significant difference was detected between the Pro content of AMF and NM pants under drought conditions, indicating that Pro in *C. migao* was not involved in the defense regulation through osmotic regulation. It is possible that the genes that regulate protein accumulation in *C. migao* are not as sensitive, so that SP do not effectively regulate the permeability of *C. migao* cell membranes.

Proline is a stress indicator, which protects the cell and enzymes and sustains osmoregulation [2]. Previous studies verified that the Pro content in AMF plants is much greater than in NM plants under drought conditions [79,80]. Pro can adjust the osmotic potential and redox reactions, thereby affecting energy transfer and preventing the generation of ROS that act as electron donors [82]. Furthermore, the accumulation of Pro is regarded as an energy supplement [83], which can provide nutrients to plants under abiotic stress. Previous studies found that the Pro content in AMF plants, including *Pistacia vera* [75], *P. trifoliata* [53], and *C. glauca* [54], was lower than in NM plants, which matched our results. Some scholars have considered that different plants adopt different resistance strategies when facing drought stress and have defined these plants as drought avoidant (lower Pro content) and drought resistant (higher Pro content) [84].

## 5. Conclusions

AMF inoculation (*G. etunicatum* and *F. mosseae*) exerted a positive effect on the growth and root vigor of *C. migao* under drought stress by strengthening key tolerance mechanisms and upregulating antioxidant enzyme activities and osmotic adjustment substances. In this study, all growth indicators and drought resistance of those indicators were significantly greater in plants inoculated with *G. etunicatum* compared to *F. mosseae*, regardless of inoculation method. Inoculation method had no significant effect on the drought resistance of *C. migao*. However, the M-seed method requires more AMF inoculum if seedlings of the same size are required, due to uneven germination of seeds. On the other hand, this method is more convenient in handling and may be preferred for afforestation of the rocky desertification areas in Karst regions, where variable size of seedlings may not be relevant.

Future research should be conducted in the field, which can better provide detailed and meaningful guidance of afforestation projects in Karst regions.

## Figures and Tables

**Table 1:** Effects of AMF status and inoculation method on malondialdehyde (MDA) content and superoxide dismutase (SOD), catalase (CAT), and peroxidase (POD) activities on the leaves of *C. migao* seedlings.

Inoculation Method	AMF Status	MDA (nmol·g^−1^)	SOD (U·g^−1^)	POD (U·g^−1^)	CAT (U·g^−1^)
M-seedling	*G. etunicatum*	35.88 ± 1.70 b	287.55 ± 3.96 a	411.52 ± 5.86 a	234.32 ± 6.86 a
	*F. mosseae*	41.82 ± 2.42 b	232.91 ± 12.84 c	259.08 ± 12.26 b	201.82 ± 5.56 b
	NM	80.19 ± 3.14 a	122.92 ± 3.83 e	116.94 ± 4.81 d	60.31 ± 5.68 c
M-seed	*G. etunicatum*	38.29 ± 2.35 b	258.57 ± 4.46 b	402.39 ± 3.48 a	223.60 ± 8.36 a
	*F. mosseae*	42.32 ± 1.18 b	203.19 ± 2.20 d	206.25 ± 4.17 c	192.97 ± 7.50 b
	NM	76.15 ± 3.34 a	125.96 ± 3.22 e	118.17 ± 7.25 d	64.80 ± 6.00 c
Two-way ANOVA (significance level)					
AMF		**	**	**	**
Method		ns	**	**	ns
AMF × Method		ns	*	**	ns

Different letters (a, b, c, d, e) indicate a significant difference of Tukey’s post hoc test (* *p* < 0.05; ** *p* < 0.01; ns, not significant) between all treatments. NM, non-AMF plants; *G. etunicatum*, plants inoculated with *G. etunicatum*; *F. mosseae*, plants inoculated with *F. mosseae*; M-seedling, inoculated by the seedling method; M-seed, inoculated by the seed method. Values are expressed as the mean ± SE (*n* = 6, which are treatment replicates).

**Table 2:** Effects of AMF status and inoculation method on the proline (Pro), soluble protein (SP), and soluble sugar (SS) contents in the leaves of *C. migao* seedlings.

Inoculation Method	AMF Status	Pro (U·g^−1^)	SP (mg·g^−1^)	SS (mg·g^−1^)
M-seedling	*G. etunicatum*	0.75 ± 0.07 b	10.73 ± 0.31 a	3.42 ± 0.20 a
	*F. mosseae*	0.61 ± 0.03 b	10.31 ± 0.31 a	3.11 ± 0.34 ab
	NM	1.11 ± 0.08 a	10.17 ± 0.67 a	2.49 ± 0.23 bc
M-seed	*G. etunicatum*	0.70 ± 0.08 b	11.77 ± 0.92 a	3.00 ± 0.21 ab
	*F. mosseae*	0.65 ± 0.08 b	10.23 ± 0.36 a	3.13 ± 0.05 ab
	NM	1.16 ± 0.06 a	10.37 ± 0.34 a	2.28 ± 0.36 c
Two-way ANOVA (significance level)				
AMF		**	ns	*
Method		ns	ns	ns
AMF × Method		ns	ns	ns

Different letters (a, b, c) indicate a significant difference of Tukey’s post hoc test (* *p* < 0.05; ** *p* < 0.01; ns, not significant) between all treatments. NM, non-AMF plants; *G. etunicatum*, plants inoculated with *G. etunicatum*; *F. mosseae*, plants inoculated with *F. mosseae*; M-seedling, inoculated by the seedling method; M-seed, inoculated by the seed method. Values are expressed as the mean ± SE (*n* = 6, which are treatment replicates).

## Data Availability

Not applicable.

## Supplementary Materials

The following are available online at https://www.mdpi.com/article/10.3390/biology11020220/s1, Figure S1: Mycorrhizal colonization of *C. migao* seedlings after AMF inoculation. *G. etunicatum*, plants inoculated with *G. etunicatum*; *F. mosseae*, plants inoculated with *F. mosseae*; M-seedling, inoculated by the seedling method; M-seed, inoculated by the seed method. Figure S2: Climactic changes during the study period at the study site. Figure S3: Plant growth of *C. migao* seedlings under 6 different treatments. NM, non-AMF plants; *G. etunicatum*, plants inoculated with *G. etunicatum*; *F. mosseae*, plants inoculated with *F. mosseae*; M-seedling, inoculated by the seedling method; M-seed, inoculated by the seed method. Figure S4: Dynamic changes in the soil moisture content during the drought stress treatment. Figure S5: Root morphology of *C. migao* seedlings under 6 different treatments. NM, non-AMF plants; *G. etunicatum*, plants inoculated with *G. etunicatum*; *F. mosseae,* plants inoculated with *F. mosseae*; M-seedling, inoculated by the seedling method; M-seed, inoculated by the seed method.
