# Supplementary material for: Different Arbuscular Mycorrhizal Fungi Established by Two Inoculation Methods Improve Growth and Drought Resistance of Cinnamomum Migao Seedlings Differently
Source: Biology (Basel). 2022 Jan 29;11(2):220. doi: 10.3390/biology11020220 (PMC8869179; doi:10.3390/biology11020220)
Supplement: Supplementary file 1 [file biology-11-00220-s001.zip › biology-1512980-supplementary.pdf]

Supplementary Materials:

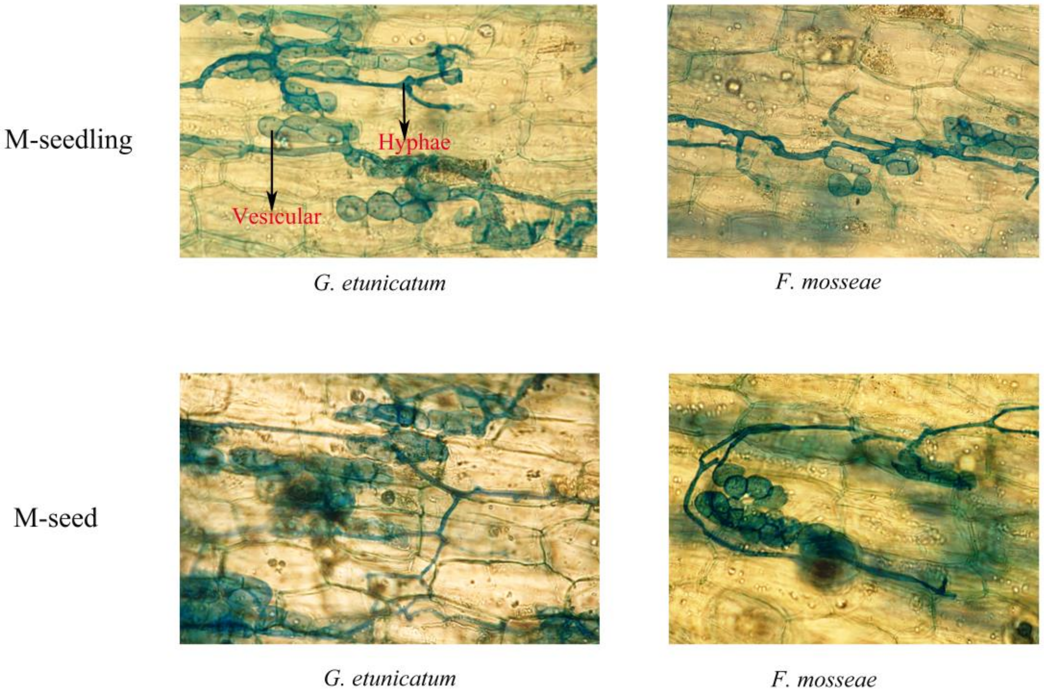

Figure S1. Mycorrhizal colonization of *C. migao* seedlings after AMF inoculation. *G. etunicatum*, plants inoculated with *G. etunicatum*; *F. mosseae*, plants inoculated with *F. mosseae*; M-seedling, inoculated by the seedling method; M-seed, inoculated by the seed method.

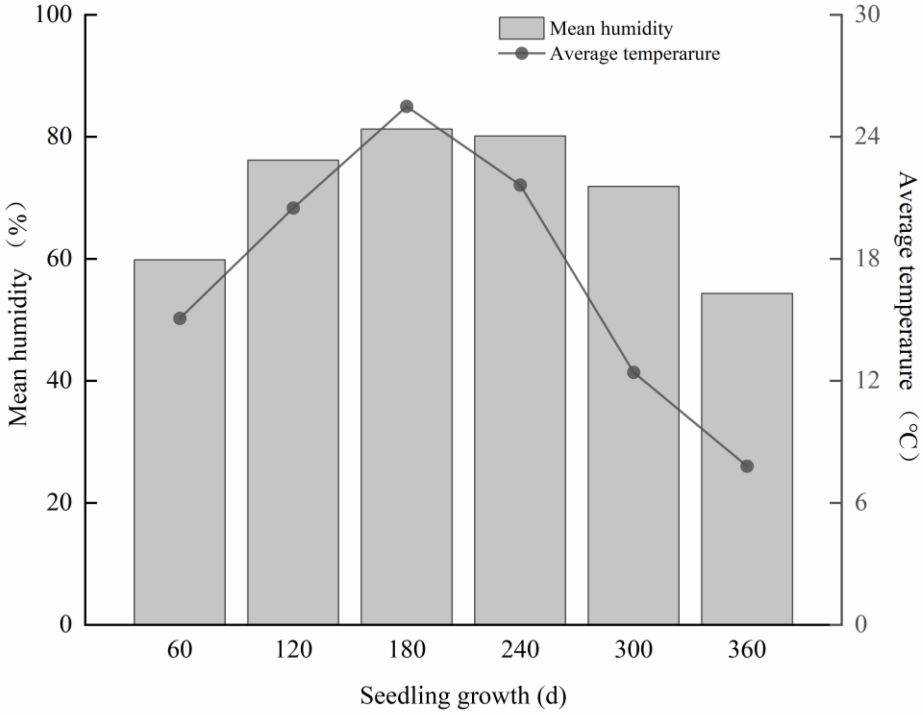

Figure S2. Climactic changes during the study period at the study site.

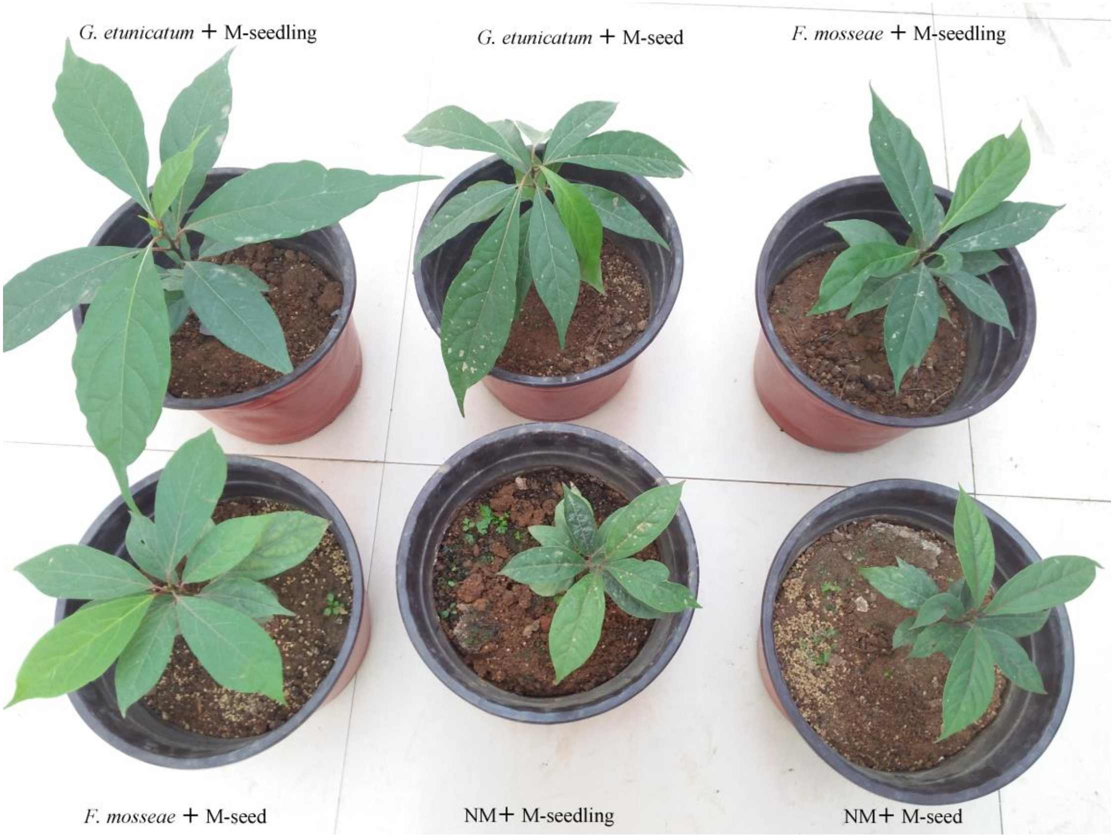

Figure S3. Plant growth of C. migao seedlings under 6 different treatments. NM, non-AMF plants; G. etunicatum, plants inoculated with G. etunicatum; F. mosseae, plants inoculated with F. mosseae; M-seedling, inoculated by the seedling method; M-seed, inoculated by the seed method.

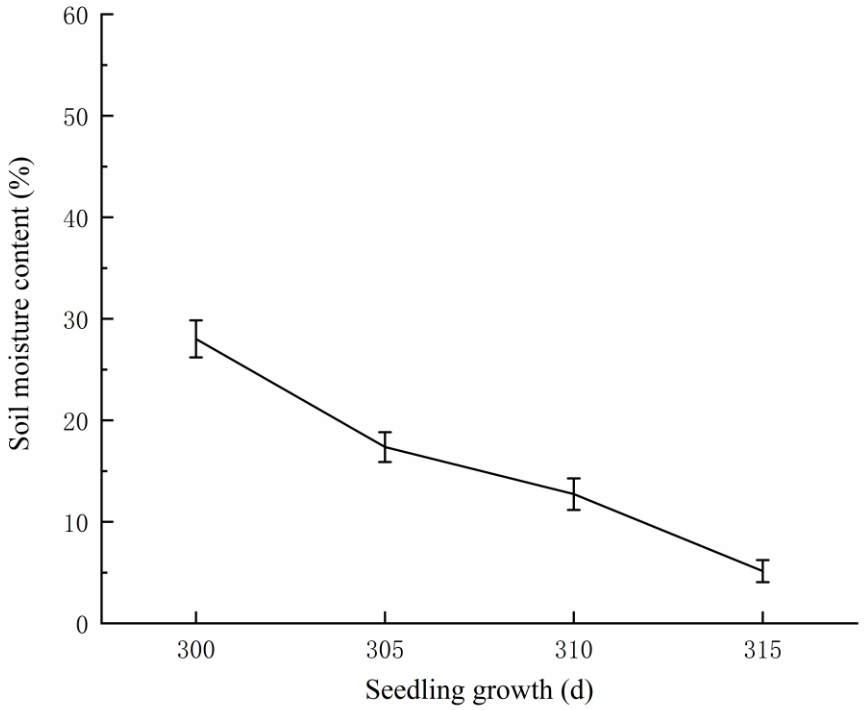

Figure S4. Dynamic changes in the soil moisture content during the drought stress treatment.

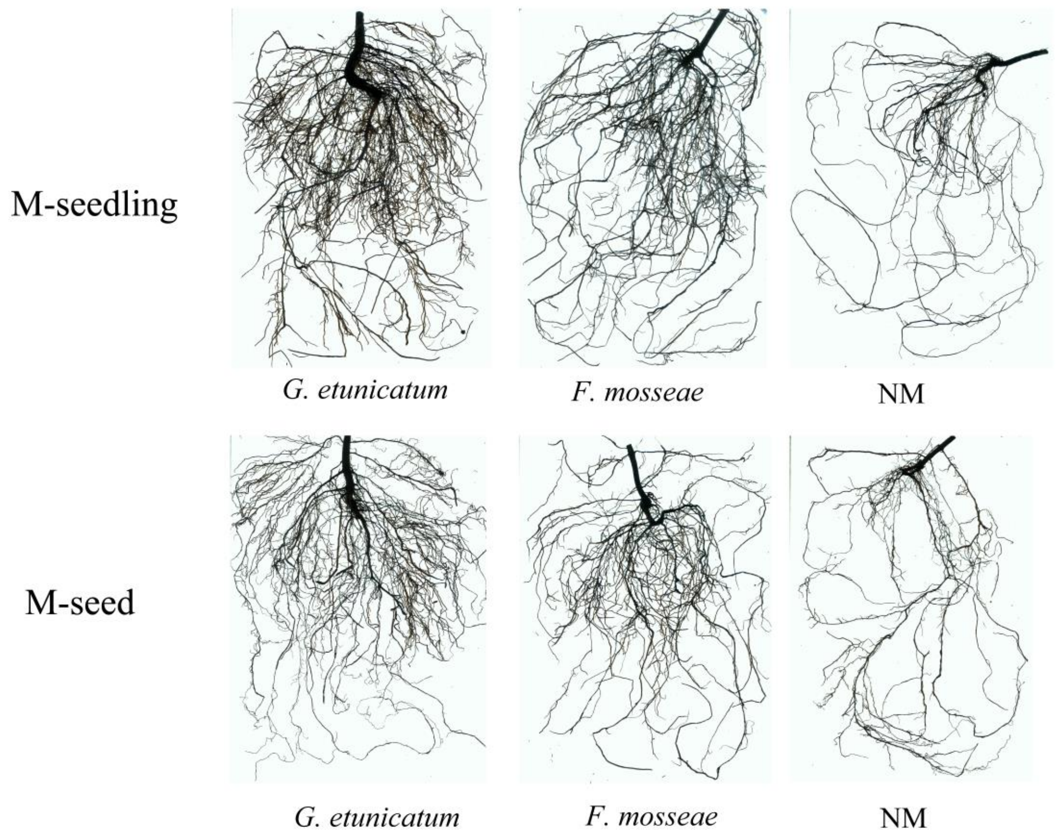

Figure S5. Root morphology of C. migao seedlings under 6 different treatments. NM, non-AMF plants; G. etunicatum, plants inoculated with G. etunicatum; F. mosseae, plants inoculated with F. mosseae; M-seedling, inoculated by the seedling method; M-seed, inoculated by the seed method.
